# From professional surveys to citizen scientists’ observations: Presence-based spatial and temporal analyses of three protected saproxylic beetles (Coleoptera, Lucanidae, Cerambycidae) in Bulgaria

**DOI:** 10.3897/BDJ.14.e192724

**Published:** 2026-05-13

**Authors:** Rumyana Kostova, Rostislav Bekchiev

**Affiliations:** 1 Sofia University, Faculty of Biology, Sofia, Bulgaria Sofia University, Faculty of Biology Sofia Bulgaria; 2 National Museum of Natural History, Bulgarian Academy of Science, Sofia, Bulgaria National Museum of Natural History, Bulgarian Academy of Science Sofia Bulgaria

**Keywords:** distribution, realised ecological niche, Natura 2000, *Lucanus cervus*, *
Morimus
*, *Rosalia alpina*, conservation

## Abstract

The saproxylic beetles *Lucanus cervus* (Linnaeus, 1758), *Morimus asper funereus* Mulsant, 1862 and *Rosalia alpina* (Linnaeus, 1758), protected under the European Habitats Directive 92/43/EEC, became the subject of targeted surveys in Bulgaria after the country began establishing its national Natura 2000 network of protected areas. Significant data accumulation began after 2011, when the mapping of the Natura 2000 sites in Bulgaria started. Records of these easy-to-recognise beetles were collected for over 20 years (since 2004) as a result of scientific surveys and citizen scientists’ posts in the Facebook group "The Insects and the Entomologists". Data have been merged into the SmartBirds platform and supplemented with data from iNaturalist available at GBIF. The database has been further enriched with historical records from literature and the National Museum of Natural History, Bulgarian Academy of Sciences collection, dating from 1897 to the present. The collated data (*L. cervus* – 1807 records; *M. asper* s.l. – 1148; *R. alpina* – 389) were summarised and analysed spatially and temporally. The present distributions of the three species by habitat type, altitude and Natura 2000 sites in Bulgaria are provided. A comparison is drawn between the data collated from both targeted monitoring surveys and amateur reports. The peaks in the seasonal dynamics of imago activity were in July for *L. cervus* (49% of the records from citizen science) and *R. alpina* (61%) and in May for *M. asper* s.l. (33%). A comparison of the record frequency before and after 2000 showed a shift to the earlier activity peak only in *M. asper* s.l. from June to May.

## Introduction

The protected saproxylic beetles *Lucanus cervus* (Linnaeus, 1758), *Morimus asper funereus* (Mulsant, 1862) and *Rosalia alpina* (Linnaeus, 1758) have been the subject of targeted surveys in Bulgaria since the country began establishing its national Natura 2000 network in 2004. These species are listed in Annex II of the European Habitats Directive 92/43/EEC and, in the case of *R. alpina*, in Annex IV as a priority species. The systematic position of the Bulgarian *Morimus* taxa — *M. asper funereus*, *M. verecundus bulgaricus* Danilevsky et al., 2016 and *M. orientalis* Reitter, 1894 (Tavakilian and Chevillotte 2025) — remains controversial (Solano et al. 2013, Gojković et al. 2022, Kostova et al. 2024). Therefore, we follow the concept proposed by Solano et al. (2013), treating them as subspecies and referring to them collectively as *Morimus asper* s.l.

Data on the occurrence of these umbrella species in Bulgaria are unevenly distributed over time. Fragmented data were reported in numerous publications prior to 2011. The earliest records for the three species in Bulgaria date back to the late 19^th^ and early 20^th^ centuries and were documented by Bulgarian naturalists, such as Yoakimov (1899), Yoakimov (1904), Markovich (1904), Markovich (1909). The most recent comprehensive summary of known records for *L. cervus* was provided by Guéorguiev and Bunalski (2004), followed by additional locality reports in Guéorguiev and Ljubomirov (2009), Guéorguiev et al. (2011), Guéorguiev (2011), Harvey et al. (2011), Cuzepan and Tăuşa (2013), Teofilova (2016), Teofilova (2017), Kostova et al. (2019). Harvey et al. (2011) compiled all known locations of *L. cervus* in Bulgaria to that time, illustrating that limited research had been conducted before targeted studies began in 2004. The most recent work summarising the known localities of *M. asper* s.l. and modelling the species’ ecological niche (Kostova et al. 2023) likewise shows that data prior to 2011 were fragmented and collected unsystematically. Later targeted research and citizen-science data resulted in a sharp increase in the number of documented sites (Kostova et al. 2023). The first synthesis of distribution data of *R. alpina* in Bulgaria was presented in Fauna of Bulgaria (Angelov 1995). More recently, fragmented data have been compiled in separate regional lists for several Bulgarian mountain ranges, including the Western Stara Planina (Georgiev 2011), Strandzha (Georgiev et al. 2018), Belasitsa (Georgiev et al. 2019), Rila (Georgiev et al. 2021), Pirin (Georgiev et al. 2022) and Vitosha (Topalov 2025) Mountains.

The European stag beetle, the rosalia linghorn and the longhorn beetle *M. asper* s.l. are easily recognisable by amateurs and are, therefore, highly suitable for citizen-science projects (Redolfi De Zan et al. 2023, Gisondi et al. 2025a). They serve as flagship species and key symbols in conservation campaigns and are amongst the most charismatic representatives of the saproxylic insects, attracting significant public interest and empathy (Drag et al. 2011, Ducarme et al. 2013, Zapponi et al. 2017, Thomaes et al. 2021, Soutinho et al. 2025). In Bulgaria, however, true citizen-science initiatives with active volunteer participation have been limited mainly to butterflies (e.g. The European Butterfly Monitoring Scheme) with no such initiatives implemented for other animal groups to date. Still, citizen-generated data have gradually begun to accumulate through so-called 'passive citizen science' (Edwards et al. 2021) via the social platforms. In 2013, the Facebook group 'The Insects and the Entomologists' was created to connect entomologists and the general public and facilitate outreach and the collection of distribution data through photographs accompanied by date and locality. The records collected through this 'passive citizen science' approach are opportunistic in nature, arising from the natural curiosity of the group participants, who post photos of their encounters with various insects in an effort to identify organisms observed in their backyards or during nature excursions. Group members are required to provide the date and place of the observation along with the photograph, as specified in the group rules they accept. Our experience has shown that requiring precise coordinates is often impractical, as some contributors perceive even requests for date and location as concerns related to personal data. Specialists on different insect groups (both professional scientists and amateurs) provide identification to the lowest possible taxonomic level and often give information about the biology, ecology, and medical, veterinary, or agricultural significance of the observed insect, making the exchange of information beneficial for both sides. As the group grew — reaching nearly 70 000 members till December 2025 — the frequency of reports of the studied beetle species likewise increased.

The initiation of systematic studies with the Natura 2000 mapping project (Project DIR-59318-1-2 2007-2013 field data 2011–2012) and subsequent development of the monitoring scheme (Project DIR-5113024-1-48 2013-2014, field data 2013–2014) led to the addition of an invertebrate database within the SmartBirds platform (Popgeorgiev et al. 2015), which was initially populated by experts. The platform enables the integration of literature records, monitoring and survey data and single observations. This established an extensive records database for the protected beetle species in Bulgaria. Building on this comprehensive dataset, understanding the environmental drivers of species distributions becomes a key next step.

The environmental conditions governing species distributions vary in their relative influence across regions, a pattern reflected in the differential importance of climatic variables within species distribution models (SDMs). This variability is well demonstrated by the distinct SDMs developed for Cerambycidae, revised by Ortega-Huerta et al. (2025). For Bulgaria, the only saproxylic beetle for which an SDM has been created is *M. asper* s.l. (Kostova et al. 2023). To date, there has been no in-depth study of the environmental factors determining the distribution patterns in Bulgaria of *L. cervus* and *R. alpina*, the relationship with the characteristics of their habitats and the degree of overlap in the distribution of the three emblematic saproxylic beetles in the country.

The aims of this study were to analyse recent spatial distribution of *L. cervus*, *M. asper* s.l. and *R. alpina*, to evaluate the dependency between the frequency of species records and their ecological preferences and to compare their seasonal dynamics by contrasting data collected by professionals and citizen scientists in Bulgaria.

## Material and methods

Presence data for *L. cervus*, *M. asper* s.l. and *R. alpina* were compiled from published literature, scientific studies (fieldwork on projects connected with protected areas and species management plans, as well as with reporting under art. 17 of the Habitats Directive (92/43/EEC)), incidental expert observations, the collection of the National Museum of Natural History-Sofia, Bulgarian Academy of Science (NMNHS) and records reported in the Bulgarian Facebook group 'The Insects and the Entomologists', covering the period from one of the first faunistic publications for the country (Yoakimov 1899) through 2025. These records were incorporated into the SmartBirds platform (https://smartbirds.org, Popgeorgiev et al. (2015)) and now are available at GBIF (https://doi.org/10.15468/scsk9u, Popgeorgiev et al. (2026)). We further supplemented the dataset with all available iNaturalist observations of the studied beetle species from Bulgaria, licensed under Creative Commons and retrieved via the Global Biodiversity Information Facility (GBIF) to the dataset (GBIF.org 2025a, GBIF.org 2025b, GBIF.org 2025c). In many cases, iNaturalist records exhibited varying levels of spatial uncertainty, with coordinate imprecision reaching up to 30 km (likely due to intentional obscuring). Such uncertainty renders these data unsuitable for ecological niche analyses. For example, during the study period, *R. alpina* records in GBIF originating from iNaturalist comprised eight records with 5 km uncertainty, 18 records with 30 km uncertainty and four records with unknown uncertainty (GBIF.org 2025c). Similar issues exist with records from 'The Insects and the Entomologists'. According to the group rules, at least the nearest populated place and the date of observation should be provided together with the photograph. Then coordinates were assigned with an accepted uncertainty of up to 5 km and the records were filled into the SmartBirds platform. Nevertheless, some records from both platforms had very precise location data.

All available data were employed to generate comparative distribution maps and to assess seasonal activity patterns. For estimating habitat use, only records with precise geographic coordinates (expert records and selected iNaturalist observations with uncertainty up to 50 m) were included. To characterise selected ecological variables representing the realised niche of the studied beetle species, occurrence records were intersected with several spatial layers: forest age and forest stand taxation derived from the Forest Database of the Forest Executive Agency (2000); altitude from the European Digital Elevation Model v.1.1 (Copernicus Land Monitoring Service 2016); and the spatial coverage of Sites of Community Interest (SCI) from Natura 2000 data (European Environment Agency 2024). All spatial analyses were conducted using QGIS 3.34 (QGIS Development Team 2024). As the Forest Executive Agency (FEA) database includes data only from Bulgarian forest enterprises, analyses were restricted to the locations available within this dataset (1025 points for *L. cervus*, 527 for *M. asper* s.l. and 115 for *R. alpina*).

To test the differences between the proportions of records for the seasonal dynamics, the Z-test incorporated in SigmaPlot 12 (Systat Software 2010) was used. A significant difference was observed when p < 0.05.

## Results

For the first time, an exhaustive dataset combining scientific and citizen observation data for *L. cervus* and *R. alpina* in Bulgaria was analysed. We included 1807 records for *L. cervus*, 1148 for *M. asper* s.l. (including 89 new records after Kostova et al. (2023), collected mostly by citizen science) and 389 for *R. alpina* (Table 1, GBIF.org 2025a, GBIF.org 2025b, GBIF.org 2025c, Popgeorgiev et al. 2026).

**Figure 1.**
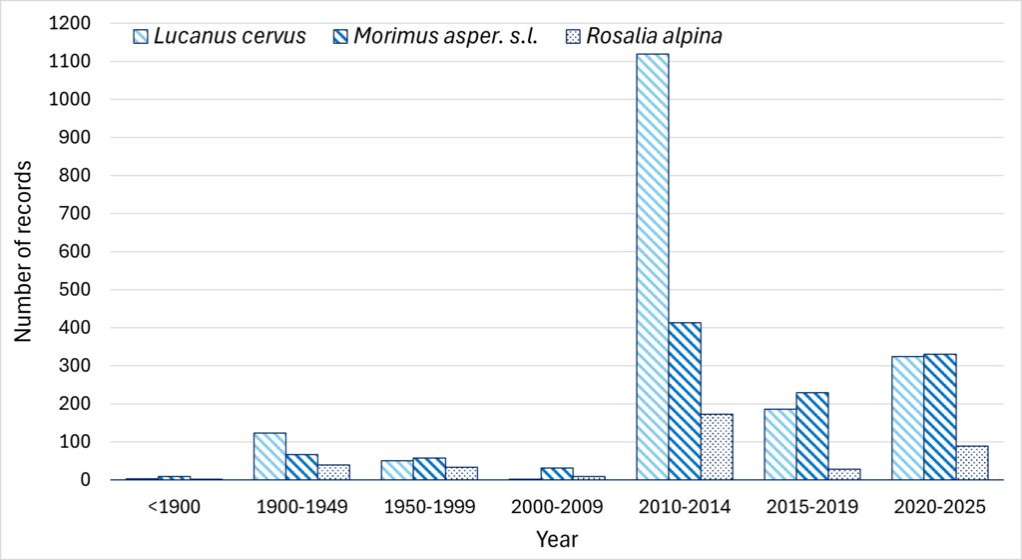
Records distribution of *L. cervus*, *M. asper* s.l. and *R. alpina* in Bulgaria over time.

The records before 2011 represent only 14% of the total records in the obtained dataset. Most of them were from the NMNHS collection (66%) and from literature (29%) (Popgeorgiev et al. 2026). With the first targeted project for collecting data on protected species under the Habitats Directive, including *L. cervus*, *M. asper* s.l. and *R. alpina* (Project DIR-59318-1-2 2007-2013), the presence records for the three species increased greatly (Fig. 1).

**Figure 2.**
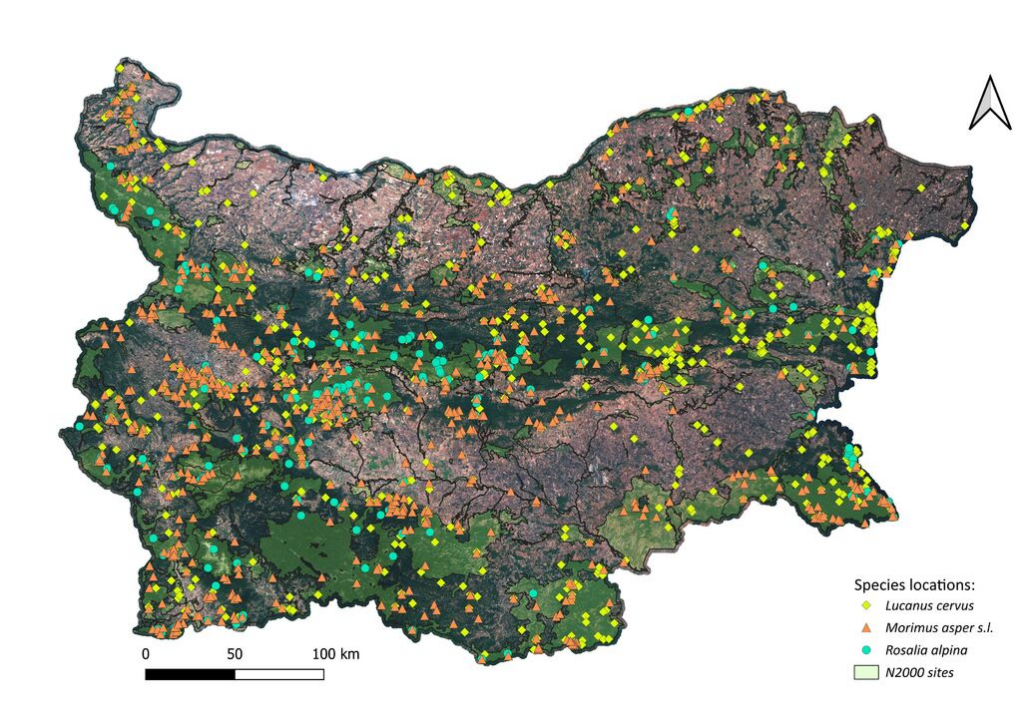
Distribution of *L. cervus*, *M. asper* s.l. and *R. alpina* and coverage of the Natura 2000 network in Bulgaria. Base: satellite map of Bulgaria, Sentinel 2 (Copernicus.eu), by Geopolymorphic Cloud.

**Figure 3.**
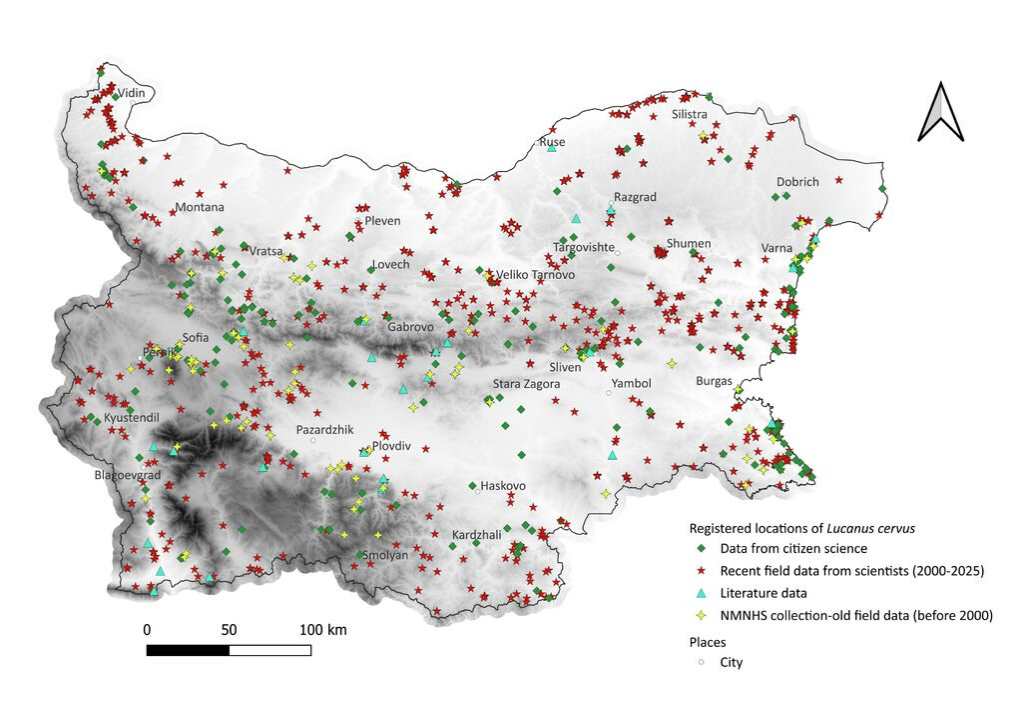
Localities of *Lucanus cervus* in Bulgaria (1898–2025). Base: Digital Elevation Model, Copernicus Land Monitoring Service (2016).

**Figure 4.**
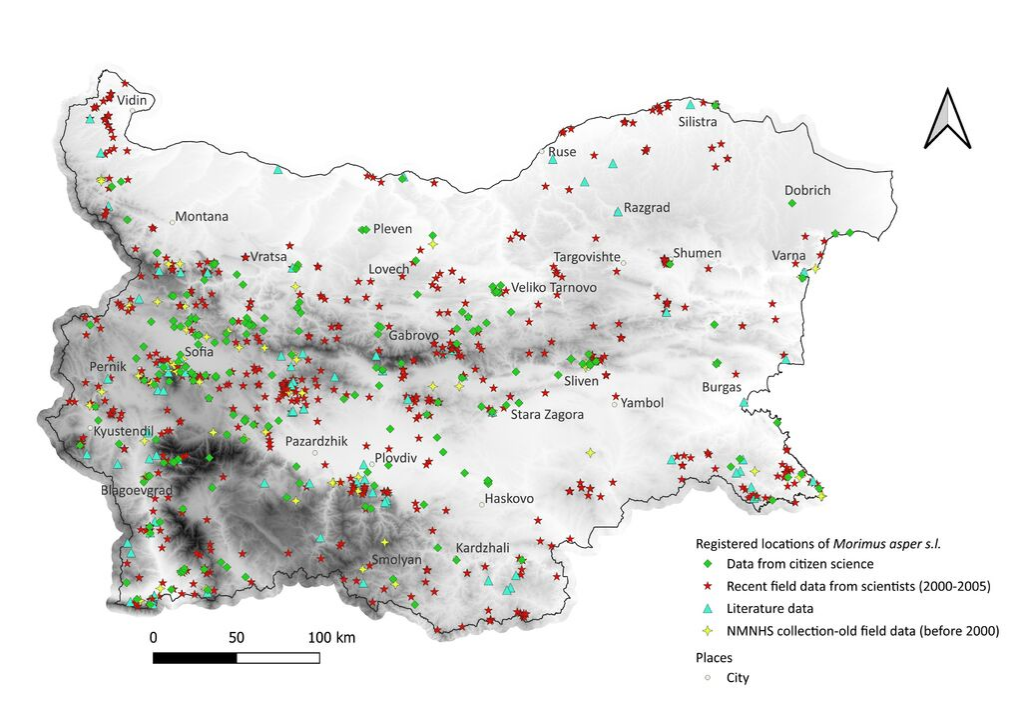
Localities of *Morimus asper* s.l. in Bulgaria. 89 records added to Kostova et al. (2023). Base: Digital Elevation Model, Copernicus Land Monitoring Service (2016).

**Figure 5.**
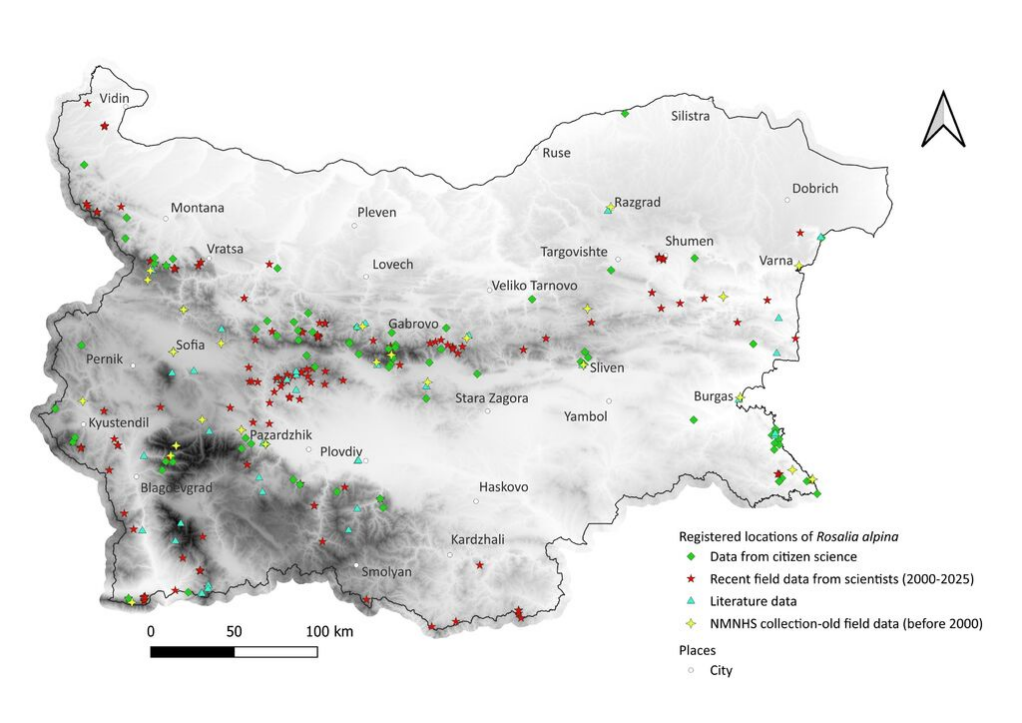
Localities of *Rosalia alpina* in Bulgaria. Base: Digital Elevation Model, Copernicus Land Monitoring Service (2016).

We mapped the occurrences of *L. cervus*, *M. asper* s.l. and *R. alpina* (Figs 2, 3, 4, 5), providing an overview of their distribution in Bulgaria. The rosalia longhorn has a restricted range, largely confined to mountainous regions and lowland forests along the Black Sea coast. The other two species have broader distributions, partly overlapping with the montane habitats of *R. alpina*, while also occupying extensive lowland broadleaf forests. Forests in the lowlands are often fragmented, particularly in northern Bulgaria where they are mainly found alongside rivers. Fragmentation also occurs along the Black Sea coast. Between 62% and 73% of records for the three species occurred within the Natura 2000 network (Table 1, Fig. 2).

Recent citizen and scientific records confirmed the persistence of old NMNHS collection localities for the three species in most cases, although to a lesser extent for *R. alpina*, for which some records around Sofia have not been confirmed (Figs 3, 4, 5).

**Altitudinal distribution of records**

**Figure 6.**
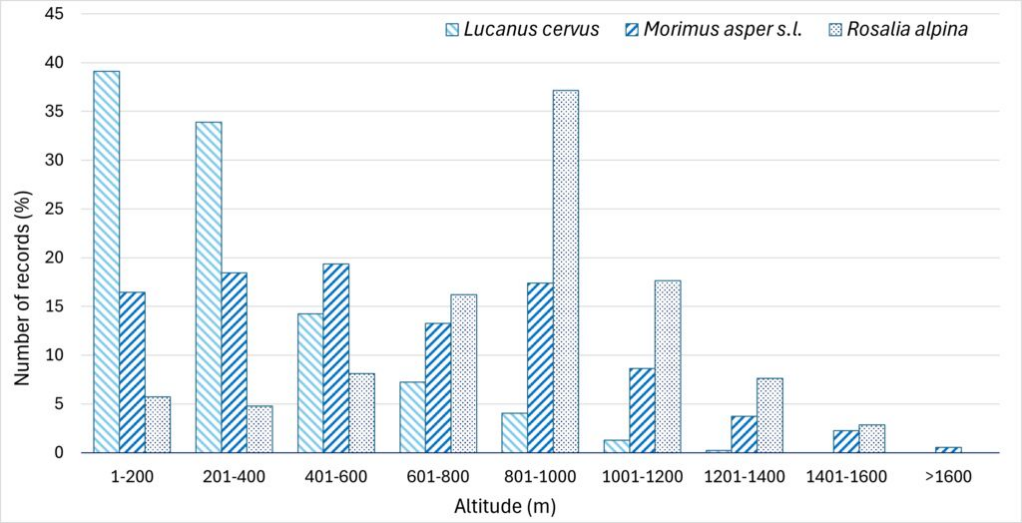
Altitudinal distribution of *L. cervus*, *M. asper* s.l. and *R. alpina* in Bulgaria.

The records of the three species demonstrated distribution in the forest belts of different altitudes. *Lucanus cervus* is mostly distributed in the lower altitude as 93% of records were found within the 1–800 m a.s.l. interval, with most of these being found between 0 and 400 m. *Morimus asper* s.l. showed more equal distribution amongst the altitude belts in the 1–800 m range, with nearly 85% of records falling within this range. *Rosalia alpina* predominantly inhabits the higher altitudes, with nearly 40% of the records between 800 and 1000 m a.s.l. and 71% between 600 and 1000 m a.s.l. (Fig. 6).

**Distribution of the records of the three species along the forest age gradient**

**Figure 7.**
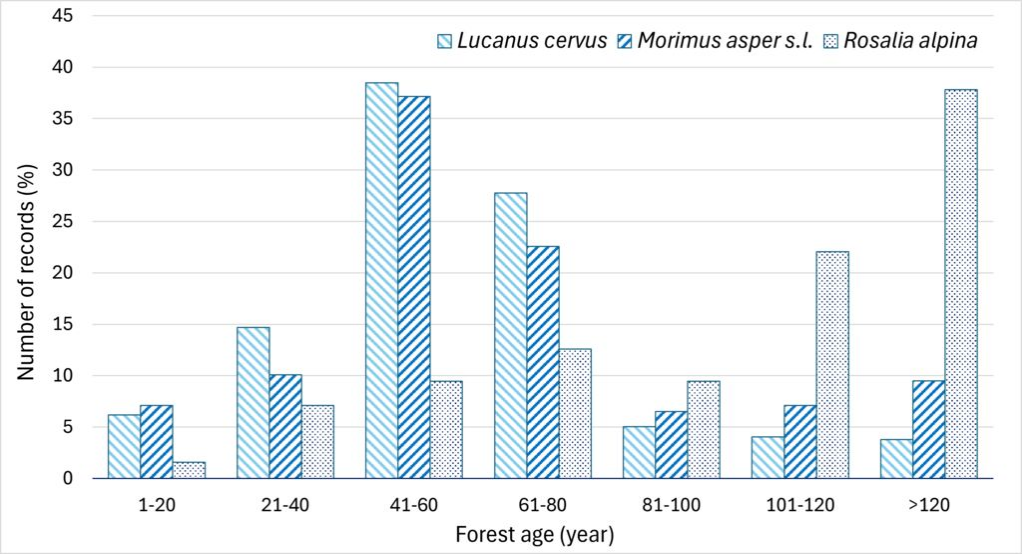
Distribution of the records of *L. cervus*, *M. asper* s.l. and *R. alpina* in Bulgaria amongst forest ages.

*Lucanus cervus* and *M. asper* s.l. were recorded not only in old stands, but also frequently in younger forests, with most records (66% for the former and 60% for the latter) occurring in stands aged 40–80 years. In contrast, 60% of *R. alpina* records were from forests older than 100 years (Fig. 7).

**Distribution of the records of the three species by forest type**

**Figure 8.**
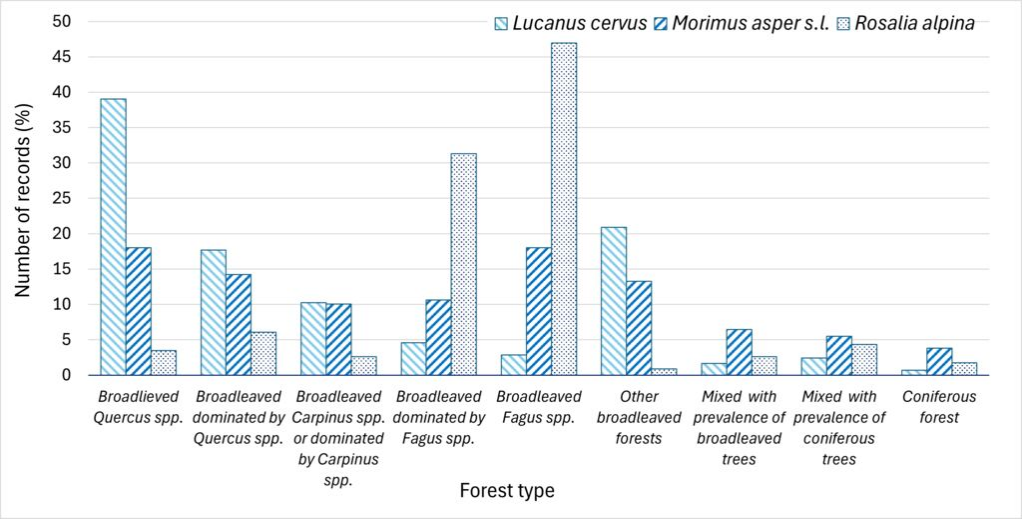
Distribution of the records of *L. cervus*, *M. asper* s.l. and *R. alpina* in Bulgaria amongst forest types.

The studied saproxylic beetles differed markedly in their preferred forest types. The European stag beetle was most frequently recorded in forests dominated by *Quercus* spp. and other deciduous trees: 39% of records occurred in pure oak forests, 18% in broad-leaved forests with oak dominance above other tree species and 21% in forests dominated by broad-leaved species other than oak, beech or hornbeam. Records from coniferous and mixed forests accounted for less than 3% of occurrences. *Morimus asper* s.l. was most commonly found in forests dominated by *Fagus* and *Carpinus* spp. (39% of records), but also occurred frequently in *Quercus* forests (32%); overall, 36% of records were evenly distributed between clear beech and clear oak forests. Approximately 10% of records for this species were from coniferous and mixed forests. *Rosalia alpina* showed a strong association with pure beech forests or stands dominated by *Fagus* spp., which accounted for 78% of records. Fewer than 10% were recorded in oak-dominated forests, around 4% in mixed beech–coniferous forests and less than 2% in other broad-leaved forest types (Fig. 8).

**Seasonal dynamics**

**Figure 9.**
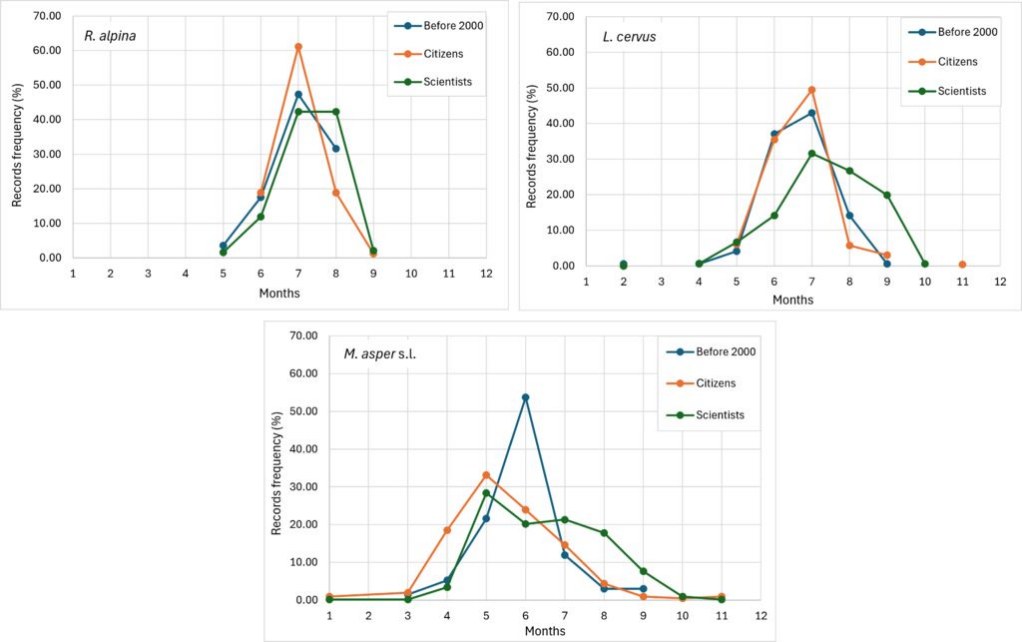
Seasonal dynamics of the record frequencies before and after 2011 (based on citizen-science and scientists’ data), for *L. cervus*, *M. asper* s.l. and *R. alpina* in Bulgaria.

The seasonality in the frequence of the records from citizen-science platforms match the phenology of *L. cervus*, *M. asper* s.l. and *R. alpina* recorded by scientists Fig. 9). Still, compared with opportunistic and targeted records collected by scientists, some differences occur in the months surrounding peak imago activity. The scientists' dataset indicated a longer activity period than the citizen-science data (*Fig. 9*). A comparison was also made between seasonal changes in record frequency from the past century and those from 2000–2025, the latter is a period during which climate change has intensified substantially and from which 98% of records were collected after 2011. The analysis did not reveal any significant shift in imago activity for the *L. cervus* and *R. alpina* in Bulgaria; for both, the peak was in July, while *M. asper* demonstrated significant shift from June to May (z = 3.65, p < 0.001), starting to increase its imago activity as early as April like the citizen data showed, confirmed by scientists’ data (z = 5.68, p < 0.001).

## Discussion

The dynamics of the records *of L. cervus*, *M. asper* and *R. alpina* in Bulgaria over the years (Fig. 1) reflect the level of research effort devoted to these beetles. Prior to 2010, there were relatively few precise records, as the species were considered to have a broad distribution and was, therefore, of limited interest for faunistic studies. The most intensive efforts occurred during the mapping of Natura 2000 sites, which corresponds to the observed peak in records. Subsequently, studies became more irregular and were conducted on a much smaller scale, mainly within monitoring projects and in relation to Natura 2000 objectives. Since 2020, records shared on the Facebook platform by participants of the group ‘Insects and entomologists’ have also been taken into account, complementing the data collected by scientists.

The distribution of records indicates partly overlapping realised ecological niches of the studied beetles in Bulgaria. *Lucanus cervus* and *M. asper* s.l. exhibit broader distributions and were recorded more frequently than *R. alpina*. Following the altitudinal distribution of forest belts in Bulgaria (Velchev et al. 1982), all three species occur mainly in broadleaved forests, with only a small proportion of records from mixed or predominantly coniferous forests (Fig. 8). About 31% of all forest habitats in Bulgaria are included in Natura 2000 sites (Tzonev and Gussev 2015). More than 60% of the records of the studied saproxylic beetles fall within these areas, indicating that a substantial proportion of their populations is covered by the network. The main habitats preferred by these species — beech and oak forests — can be considered well represented, as all forest habitat types exceed the 10% gap threshold proposed by Rosati et al. (2008). However, ongoing threats, such as legal and illegal logging, infrastructure development and natural pressures including climate change and wildfires, place the future prospects of Bulgarian forests in an unfavourable position. Natura 2000 alone, therefore, remains insufficiently effective for conserving forest habitats in Bulgaria and the establishment of additional strictly protected areas is needed (Tzonev 2011). Furthermore, D'Amen et al. (2013) showed that Natura 2000 sites designated primarily based on vegetation may be less effective in covering invertebrate diversity, particularly saproxylic beetles. Moreover, the network ensures relatively good connectivity and protection mainly in the southern parts of the country (Fig. 2). At the national scale, it likely does not provide sufficient connectivity amongst habitats suitable for the studied species, particularly along the Black Sea coast and in the lowland forests of northern Bulgaria and the Thracian Valley, which are continuously declining due to human activity (Biserkov et al. 2015). Habitat fragmentation and the lack of ecological corridors pose a critical threat to the survival and genetic diversity of *L. cervus*, *M. asper* s.l. and *R. alpina*, given their limited dispersal capacities (Bosso et al. 2013). Although capable of flight, males of *L. cervus* can disperse up to 3 km and females up to 1 km (Rink and Sinsch 2007, Tini et al. 2017, Thomaes et al. 2018), while *R. alpina* can reach distances of up to 1.5 km (Drag et al. 2011). In contrast, the wingless *M. asper* s.l. has a much more restricted dispersal range, typically up to 500 m (Rossi de Gasperis et al. 2016). These limitations highlight the need to develop targeted strategies for the conservation and restoration of ecological corridors for saproxylic species, particularly in the most affected regions of Bulgaria (Kostova et al. 2023).

One of the factors influencing the distribution pattern of the European stag beetle, rosalia longhorn and *M. asper* s.l. is the nutritional requirements of their larvae. Although larvae of all three species are considered polyphagous, they exhibit distinct host-tree preferences (Harvey et al. 2011, Bărbuceanu et al. 2015, Campanaro et al. 2017a, Leonarduzzi et al. 2017). *Morimus asper* and *L. cervus* utilise a broader spectrum of host trees. *Lucanus cervus* has been reported to develop in *Quercus*, *Fagus*, *Salix*, *Populus*, *Tilia*, *Aesculus*, *Ulmus*, *Pyrus*, *Prunus*, *Fraxinus*, *Castanea*, *Alnus* and *Pinus* (Franciscolo 1997, Harvey et al. 2011). *Morimus asper* has been recorded developing in *Fagus*, *Abies*, *Quercus*, *Acer*, *Alnus*, *Castanea*, *Platanus*, *Juglans*, *Populus*, *Prunus*, *Salix*, *Ulmus*, *Tilia*, *Carpinus*, *Robinia pseudoacacia*, *Pseudotsuga menziesii*, *Picea* and *Pinus* (Jurc et al. 2008, Hardersen et al. 2017, Doychev et al. 2017). In contrast, the rosalia longhorn shows more pronounced preferences, mainly for *Fagus* spp., but may also develop in *Acer*, *Ulmus*, *Fraxinus*, *Castanea*, *Alnus*, *Salix*, *Corylus*, *Tilia* and *Carpinus* (Migliaccio et al. 2004, Campanaro et al. 2017a). In accordance with larvae requirements, our results indicate that *L. cervus* was most frequently recorded in the lower altitudinal belts within xerothermic oak forests and mesophilic to xeromesophilic oak–hornbeam forests in Bulgaria, as well as mixed broadleaved forests. Records of *R. alpina* revealed a predominantly montane distribution, with the species largely confined to higher-elevation beech forests. In contrast, *M. asper* showed the widest altitudinal range, occurring primarily in beech and oak forests from lowland areas to the higher mountain beech belt. The distributions of the three species overlapped in mesophilic beech and oak forests of the lower mountain zones of Belasitsa, Rila, Sredna Gora, Vitosha etc. and in non-mountain areas, such as Shumen Plateau. An unusual co-occurrence was also observed in an otherwise drier region of the Eastern Rhodopes. In Valchi Dol Nature Reserve, an inversion of the oak and beech forest belts occurs, with beech forest confined to a small humid valley surrounded by dry oak forest; all three species (*L. cervus*, *M. asper* s.l. and *R. alpina*) were recorded within this beech stand. The studied beetles were also found together in lowland forest habitats along the Black Sea coast. During the Late Quaternary glaciations, the Black Sea coastal region functioned as an important refugium, supporting clear and mixed oak forests, beech forests and flooded “longoz” forests (Filipova-Marinova et al. 2024). At present, these habitats are amongst the most threatened in Bulgaria, experiencing increasing fragmentation due to clearcutting and the expansion of tourism infrastructure (Biserkov et al. 2015). An important contact zone for the three species is also the humid Tertiary forests of the Strandzha Mountains, dominated by oriental beech and various oak species. There, even within a small area — for example, in the Silkosia Reserve and in the surrounded forest near the village of Kosti — all three species can be found, together with the additional umbrella species *Cerambyx cerdo*, which is also one of the emblematic protected saproxylic beetles (authors’ data). The absence of glaciation during the Quaternary glacial periods, which allowed the region to function as a Tertiary refugium, together with its transitional biogeographic position between the Armenian Highlands and Lesser Caucasus, through the Pontic Mountains of the Anatolian Peninsula towards continental Europe, has contributed to the isolation of plant populations and the formation of habitats unique to Bulgaria (Savev et al. 2015). In this region, *M. asper* s.l. represents a distinct mitochondrial lineage compared to all other European and Asian populations (Kostova et al. 2024). Furthermore, with the exception of the Strandzha forests, coastal forests along the Black Sea preserve the Anatolian COI lineage of this beetle, which is very rare in the inland Balkans (Kostova et al. 2024). Therefore, the Black Sea coastal forests, including those in Strandzha, are of considerable conservation importance for preserving the genetic diversity of *M. asper* s.l. and likely also for the other two species, although their populations in Bulgaria have not yet been genetically studied.

As saproxylic, all three umbrella beetle species depend on dead wood, which explains their preference for old-growth forests (Bardiani et al. 2017, Campanaro et al. 2017a, Hardersen et al. 2017). Our data confirm the preference of *R. alpina* in forests older than 100 years. This persistent pattern is likely related to larval requirements for large-diameter standing trunks, which provide more stable conditions in terms of food availability and humidity (Castro et al. 2012, Campanaro et al. 2017a). In contrast, *M. asper* and especially *L. cervus* showed greater tolerance to forest age and were frequently recorded in much younger forests (Fig. 7). However, this also reflects the larger area of younger forests at low altitudes. This may be partly explained by the substantially reduced extent of old forests due to systematic deforestation in Bulgaria’s low-altitude woodlands, where secondary coppice forests now predominate, but dead wood is still present. Nowadays, coppice forests in Bulgaria occupy about 48% of the country's forest areas, mainly in the lowlands, hills and foothills. Within this coppice forest area, oak species predominate (60% of the area), followed by beech (10%), acacia (9%), downy oak (8%) and hornbeam (6%) (Trichkov and Marinova 2020). Although it is well established in literature that the preferred forest age for the European stag beetle is generally over 70 years, the species is known to develop even with much smaller amounts of dead wood, including in wood chips, fences, wooden posts or railroad ties (Campanaro et al. 2017b, Méndez and Thomaes 2021). This ecological flexibility likely explains its ability to successfully occupy younger forests (40–60 years), where it predominates in Bulgaria, as shown by our results. A similar pattern applies to *M. asper* s.l., which has also been recorded developing in coppiced stands with the presence of dead wood (Hardersen et al. 2017).

Although targeted citizen science-projects dedicated to monitoring *L. cervus*, *M. asper* s.l. or *R. alpina* have been successfully conducted in some European countries (Campanaro et al. 2017b, Thomaes et al. 2021, Redolfi De Zan et al. 2023, Gisondi et al. 2025a), such initiatives have not yet been implemented in Bulgaria. Nevertheless, our results show that opportunistic observations from 'passive citizen science', such as those collected via a Facebook group, can also provide valuable data on species distribution. These records tend to cover urbanised environments and their surroundings, whereas scientific research has traditionally focused on specific natural habitats and protected areas (Figs 3, 4, 5). Notably, even in targeted citizen-science programmes, volunteer transects are often established near populated areas, in locations convenient for participants (Thomaes et al. 2021, Gisondi et al. 2025b) These observations can also provide reliable data on imago activity. Even though the peak activity of beetles varies over the years and is highly dependent on climatic factors in the spring months of a given year (Méndez et al. 2017), the frequency of records over longer periods of time can give an idea of the general phenology of the species. The longer activity period shown by the scientific data relative to the citizen-science observations (Fig. 9) is largely explained by the inclusion of records based on dead specimens. Most Bulgarian monitoring programmes to date have been conducted outside or towards the end of the optimal season for these three species, which likely inflated the apparent duration of activity in the scientific records. A shift of peak activity to earlier periods and a shortening of the activity period due to the intensification of climate change in recent years were expected for all three species. For example, Wembridge et al. (2025) demonstrated a shortening of the imago activity period of *L. cervus* between 1998 and 2022 in the United Kingdom. However, no such shift was observed for *L. cervus* or *R. alpina* in the present study. Only *M. asper* s.l. appeared to respond to climate change by shifting its peak activity period from June to May (Fig. 9). This pattern was confirmed by both citizen-science data and data collected by professional researchers. The lack of a response in the rosalia longhorn was easier to explain, as its preferred beech forest habitat likely retains sufficient moisture and provides adequate cooling under Bulgarian climatic conditions. In contrast, the European stag beetle appeared to be more tolerant to recent climatic changes, characterised by cool and very wet springs followed by extremely hot and dry summers in the lowlands. Furthermore, climate change is expected to substantially affect the forest habitats of these three species. Compared with the 1961–1990 reference period, Bulgaria shows a clear warming trend and altered precipitation regimes, with more frequent torrential rainfall and hot, dry periods (Bocheva et al. 2023). Forests between 0 and 800–900 m a.s.l., particularly in the Continental–Mediterranean zone, are especially vulnerable, with sensitive *Quercus* species and beech foothill ecosystems likely to be most affected (Raev et al. 2015). The downy oak (*Q. pubescens* Willd.), which is the most drought-resistant species of oak found in the country will be of great importance in the adaptation of forests to climate change, but, for now, it is only found in small areas (Trichkov and Marinova 2020). Climate models further predict reduced forest productivity in the upper beech belt, with productivity decline ranging from approximately 20% to over 50% by 2090 under different scenarios. These losses are expected to be most pronounced near the southern distribution limit of *Fagus sylvatica*, where persistent high-pressure atmospheric systems are likely to intensify drought conditions (Martinez del Castillo et al. 2022). To achieve greater sustainability of forest ecosystems to climate change, the establishment of mixed forest composition phytocoenoses is recommended (Stoyanova and Stoyanov 2012), along with the overall maintenance of high biodiversity levels in forest ecosystems and high genetic diversity within tree populations (Zhelev and Aneva 2018). The measures should be evaluated also taking into consideration the saproxylic communities and their tolerance to the changing habitats.

While the presented results provide important information on the distribution, habitat use and phenology of three saproxylic beetle species of community interest, they also clearly demonstrate that, despite more than 20 years of contemporary monitoring efforts, current knowledge on their population density and ecological responses remains insufficient — even for common and relatively easy-to-observe species such as the stag beetle, the rosalia longhorn and *M. asper*. Similar results have been reported in targeted long-term programs combining citizen science and professional efforts across Europe, where, even for the 'distribution' parameter, full coverage was not achieved (Thomaes et al. 2021, Gisondi et al. 2025b). As the number of entomologists in Bulgaria is declining for various reasons, the only short-term solution for obtaining reliable information on these species is to strengthen citizen science, conduct training and introduce a monitoring scheme covering the entire territory of the country. In this regard, it would be important to involve the numerous teachers and students, who are located in almost every part of the country and often show high-levels of enthusiasm and skills in studying nature. Involving volunteers in targeted programmes would not only draw attention to saproxylic insects — which are less popular than mammals and birds, for example — but also engage the general public in the conservation of forests and their inhabitants in Bulgaria. Data collection must be carried out using evidence-based applications (SmartBirds, iNaturalist) that allow for the collection of accurate and standardised data.

Effective conservation of *L. cervus*, *M. asper* and *R. alpina* requires both the preservation of existing populations and the maintenance of functional connectivity amongst forest habitats, at least within the Natura 2000 network. Molecular approaches, including genomics and phylogenetics, can provide essential insights to support the conservation of these species and the associated forest ecosystems. Their application is especially relevant in light of persistent taxonomic uncertainties within the genus *Morimus* Brullé, 1832 (Solano et al. 2013, Gojković et al. 2022, Kostova et al. 2024) and even within *Lucanus* Scopoli, 1763 (Solano et al. 2016) in Europe, which pose challenges for the conservation management. Available genetic evidence indicates that these ambiguities may be attributable to mitochondrial introgression (Solano et al. 2013, Solano et al. 2016, Gojković et al. 2022, Kostova et al. 2024). Furthermore, *R. alpina* also demonstrates substantial genetic diversity, particularly in the southern parts of its distribution range compared to Central European populations (Drag et al. 2015, Molfini et al. 2018). In this context, the delineation of management units, based on well-supported clades exhibiting significant genetic divergence, represents a robust basis for conservation planning (Drag et al. 2011, Solano et al. 2013, Gojković et al. 2022, Kostova et al. 2024).

## Figures and Tables

**Table 1.** Number of records obtained from different sources within the dataset.

Species	Records source					Registered within Natura 2000 sites		
	Published literature	NMNHS collection	Scientific studies	Citizen science		N records	N sites	% of total records
				FB group	iNaturalist			
*L. cervus*	28	176	1338	97	168	1308	116	72.39
*M. asper* s.l.	96	83	764	90	115	718	107	62.54
*R. alpina*	41	51	215	54	28	280	44	71.97
